# Electrochemical DNA Sensor Based on the Copolymer of Proflavine and Azure B for Doxorubicin Determination

**DOI:** 10.3390/nano10050924

**Published:** 2020-05-10

**Authors:** Anna Porfireva, Gennady Evtugyn

**Affiliations:** 1 A.M. Butlerov’ Chemistry Institute of Kazan Federal University, 18 Kremlevskaya Street, 420008 Kazan, Russia; porfireva-a@inbox.ru; 2 Analytical Chemistry Department of Chemical Technology Institute of Ural Federal University, 19 Mira Street, 620002 Ekaterinburg, Russia

**Keywords:** electropolymerization, poly(Azure B), poly(proflavine), DNA sensor, doxorubicin determination, electrochemical impedance spectroscopy

## Abstract

A DNA sensor has been developed for the determination of doxorubicin by consecutive electropolymerization of an equimolar mixture of Azure B and proflavine and adsorption of native DNA from salmon sperm on a polymer film. Electrochemical investigation showed a difference in the behavior of individual drugs polymerized and their mixture. The use of the copolymer offered some advantages, i.e., a higher roughness of the surface, a wider range of the pH sensitivity of the response, a denser and more robust film, etc. The formation of the polymer film and its redox properties were studied using scanning electron microscopy and electrochemical impedance spectroscopy. For the doxorubicin determination, its solution was mixed with DNA and applied on the polymer surface. After that, charge transfer resistance was assessed in the presence of [Fe(CN)_6_]^3−/4−^ as the redox probe. Its value regularly grew with the doxorubicin concentration in the range from 0.03 to 10 nM (limit of detection 0.01 nM). The DNA sensor was tested on the doxorubicin preparations and spiked samples mimicking blood serum. The recovery was found to be 98–106%. The DNA sensor developed can find application for the determination of drug residues in blood and for the pharmacokinetics studies.

## 1. Introduction

Electropolymerization is an advanced tool of modern electrochemistry that is frequently used for the modification of the electrodes in the assembly of electrochromic devices and electrochemical biosensors [1,2]. In these reactions initiated by primary electron transfer, oligomeric products are formed and deposited on the electrode as a dense uniform film, whose properties are derived from those of the monomers [3,4,5]. In most cases, electropolymerized products play role of heterogeneous mediators of the electron transfer or of the matrix wiring the bioreceptors and the nanomaterials (metals, carbon nanoparticles) [6,7,8] and providing the immobilization of biochemical components [9,10]. They offer many advantages over conventional modifiers used for the same purpose. Among them, one-step synthesis, controlled electrochemical activity, simple modification with various functional groups, and quantification of the electrodeposition by the current or the charge transferred are mentioned. All the polymers obtained by the electrolysis are divided into three groups, i.e., electroconductive, electrochemically active, and inactive polymers. Polyaniline [11,12], polypyrrole [13], polythiophene [14], and their derivatives are mostly used in electrochemical sensors and biosensors as electroconductive polymers. 

Polymeric forms of the phenazine and phenothiazine dyes have found application among electrochemically active polymers [15,16,17] and some functionalized polyphenols as inactive coatings. Methylene blue and Neutral red in polymeric form have been described in the assembly of the electrochemical sensors for the determination of many small organic molecules interesting for medicine, pharmacy, and environmental monitoring, e.g., catechin [18], nevirapine [19], paracetamol [20], vanillomandelic and homovanillic acids [21], catechol and hydroquinone [22], and ascorbic acid [23]. Methylene blue has found a broad application as a redox probe and diffusionally-free indicator in DNA- and aptasensors [24,25]. Neutral red was also implemented in the biosensor assembly for mediation of the electron transfer [26,27,28]. Although they show satisfactory characteristics of the electron transfer, their application can be limited by some drawbacks, e.g., very high non-specific adsorption of the Methylene blue and low selectivity of mediation by the Neutral red. 

Recently, other derivatives of the phenazine dyes (Azure A, Azure B) and proflavine able to change their electrochemical properties in the presence of the proteins and DNA have been introduced in the electroanalysis [29,30,31,32]. Thus, proflavine, an acridine dye, was successfully used for the detection of the DNA hybridization events due to the ability to intercalate double-stranded DNA helix and to influence the electron exchange conditions on the electrode interface [33]. Additionally, it was used for the assessment of the DNA melting point changed due to the ligand binding [34]. Azure B was electropolymerized on the Pt and glassy carbon electrode (GCE) in acidic media [30] and polycrystalline Au [35] showing reversible redox behavior both in monomeric and polymeric form. The only examples of the application of the polymeric proflavine and Azure B in the assembly of the DNA sensors were done in our previous works [29,31], where rather low efficiency of the electropolymerization referred to the low solubility of the monomers in neutral media.

In this work, a mixed copolymer of the proflavine and Azure B has been obtained for the first time and applied for the doxorubicin determination. It was shown that the use of the copolymer changed the mechanism of the signal formation and the permeability of the surface layer became more important than the electrostatic interactions in the doxorubicin determination. The DNA sensor developed made it possible to detect 0.01 nM doxorubicin with a satisfactory recovery demonstrated in artificial blood plasma. 

## 2. Materials and Methods 

### 2.1. Reagents

Azure B (3-(dimethylamino)-7-(methylamino)phenothiazin-5-ium chloride, 97%), proflavine hydrochloride (3,6-Diamino-10-methylacridinium chloride, 95%), doxorubicin hydrochloride ((8S,10S)-6,8,10,11-tetrahydroxy-8-(hydroxyacetyl)-1-methoxy-7,8,9,10-tetrahydrotetracene-5,12-dione, 97%), low molecular double-stranded DNA from salmon sperm (average mol. mass 4.6 kDa [36]) HEPES (4-(2-hydroxyethyl)-1-piperazineethanesulfonic acid), hydroquinone, and bovine serum albumin (BSA) were purchased from Sigma-Aldrich, Dortmund, Germany (https://www.sigmaaldrich.com/catalog). Other reagents were of analytical grade. Deionized Millipore^®^ water (Simplicity^®^ water purification system, Merck-Millipore, Molsheim, France, https://www.merckmillipore.com/) was used for the preparation of working solutions. The pH dependence of the polymer coating properties was monitored using Britton–Robinson buffer consisting of 0.04 M H_3_PO_4_, 0.04 M H_3_BO_3_, 0.04 M CH_3_COOH, 0.05 M Na_2_SO_4_). The DNA stock solutions (1 or 10 mg/mL) were prepared in 0.1 M HEPES containing 0.03 M NaCl, pH 7.0. Electropolymerization was performed in 0.025 M phosphate buffer (PB) containing 0.1 M KCl. Ringer–Locke’s solution was used for mimicking influence of blood electrolytes. It contained 0.45 g NaCl, 0.021 g KCl, 0.016 g CaCl_2_·2H_2_O, 0.005 g NaHCO_3_, 0.075 g of glucose, 0.015 g of MgSO_4_, and 0.025 g of NaH_2_PO_4_·2H_2_O per 50 mL of water [37]. Doxorubicin preparations LANS^®^ (“LANS-Verofarm”, Belgorod, Russia, https://products.veropharm.ru/en) and TEVA (“Teva Pharmaceutical Industries”, Petah Tikva, Israel, https://tevapharm.com) were purchased in the local pharmacy market. 

### 2.2. Apparatus

Voltammetric and impedimetric measurements were conducted at ambient temperature with the potentiostat-galvanostat AUTOLAB PGSTAT 302N (Metrohm Autolab b.v., Utrecht, The Netherlands, https://www.metrohm.com/en/products/electrochemistry) equipped with the FRA2 module. The electropolymerization and the DNA deposition were performed using the GCE (1.67 mm^2^) as working electrode, Pt wire as auxiliary electrode and Ag/AgCl/3 M KCl as reference electrode (Metrohm Autolab b.v.). The electrochemical impedance (EIS) spectra were recorded at the equilibrium potential with the amplitude of applied sine potential of 5 mV and the frequency varied in the range from 100 kHz to 0.04 Hz in the presence of 0.01 M K_3_[Fe(CN)_6_] and 0.01 M K_4_[Fe(CN)_6_]. The equilibrium potential was calculated as the half-sum of the cathodic and anodic peak potentials of the [Fe(CN)_6_]^3−/4−^ peak pair. The impedance parameters were determined by fitting with the equivalent circuit (*R_ct_C*)*_1_*(*R_ct_C*)*_2_*, where *R_ct_* is the charge transfer resistance and *C* the constant-phase element representing the non-ideal capacitance behavior. Indices *1* and *2* correspond to the outer and inner interfaces (electrode–film and film–solution). Equivalent circuit fitting was performed with the NOVA software (Metrohm Autolab b.v.). 

The pH measurements were performed with the EXPERT-001-1 digital pH meter-ionometer (Econix-Expert Ltd, Moscow, Russia, http://ionomer.ru/index.php?lang=english). 

Scanning electron microscopy (SEM) images were recorded on a field emission scanning electron microscope Merlin™ (Zeiss, Jena, Germany, https://www.zeiss.com/microscopy/int/products/scanning-electron-microscopes.html). The films obtained by electropolymerization were preliminarily coated with the Au/Pd layer in vacuum by a T150ES sputter coater (Quorum Technologies Ltd, Laughton, United Kingdom, https://www.quorumtech.com).

### 2.3. Electropolymerization of Azure B and Proflavine and DNA Sensor Preparation

The GCE was first mechanically polished to a mirror-like surface and cleaned with acetone and sulfuric acid. Then, it was immersed in the solution obtained by mixing 4.6 mL of 0.025 M PB, 385 μL of Azure B (1.03 mg/mL), and 22 μL of proflavine (15 mg/mL). Resulting concentrations of the dyes were equal to 0.25 mM of each. Then, 20 cycles of the potential in the range from −0.4 to 1.2 V were run with the scan rate of 100 mV/s. After that, the electrode was rinsed with deionized water. After drying, the electrode was fixed upside down and 2 μL of DNA solution (1 mg/mL) in 0.1 M HEPES containing 0.03 M NaCl, pH = 7.0, were spread on the working area. After drying, the electrode was washed several times with the PB and deionized water and used for electrochemical measurements.

### 2.4. Doxorubicin Determination

The doxorubicin solution was mixed with the 10 mg/mL DNA solution in the 9:1 ratio (*v*:*v*). Then, 2 μL of the mixture were placed on the working surface of the GCE covered with the Azure B–proflavine copolymer and dried at ambient temperature. The electrode was washed with deionized water to remove unbound reactants and transferred to the cell containing 4.5 mL of 0.025 M PB with 0.1 M KCl, pH = 7.0, and 0.5 mL of 0.1 M equimolar mixture of K_3_[Fe(CN)_6_] and K_4_[Fe(CN)_6_]. After magnetic stirring, the EIS spectrum was recorded and the Nyquist diagram plotted to determine the charge transfer resistance as a measure of the doxorubicin concentration.

## 3. Results

### 3.1. Copolymerization of Azure B and Electrochemical Properties of the Polymerization Product

**Figure 1:**
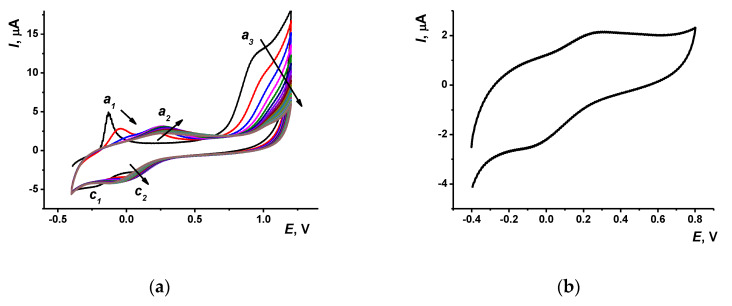
(**a**) Multiple cyclic voltammograms recorded on glassy carbon electrode in 0.025 M PB, pH = 7.0, containing 0.03 M NaCl, 0.25 mM Azure B, and 0.25 mM proflavine, scan rate 100 mV/s (20 cycles). Arrows indicate direction of the changes observed with increasing number of the cycles. (**b**) Cyclic voltammograms recorded with glassy carbon electrode covered with Azure B–proflavine copolymer (20 cycles) in 0.025 M PB with no monomers.

The cyclic voltammograms obtained in a multiple cycling of the potential in the mixture of monomeric Azure B and proflavine are presented in Figure 1. Most changes on the voltammograms have been finished to the 20th cycle. Thus, in the following experiments, the electropolymerization was performed using this number of potential cycles.

At the first scan, high anodic peak (*a_1_* on voltammogram) appears at −0.13 V. In the second cycle, it was decreased about twice and shifted to less negative potential (−0.04 V). Meanwhile, the second anodic peak (*a_2_*) appeared at −0.20 V. In the following scans, anodic peak (*a_1_*) disappeared and the second one was shifted to 0.35 V and insignificantly decreased in height.

In the opposite direction of the potential scan, changes in the peaks recorded in the same potential range were quite different. On the first scan, two small cathodic peaks appeared on voltammogram, from which one (*c_1_*) derived from the peak *a_1_* and second one (*c_2_*) was probably related to the anodic peak *a_2_* though it appeared on the next cycles. Additionally, these two pairs of reversible peaks, a high irreversible anodic peak was observed at the potentials higher than 0.93 V. This peak decreased in height and shifted to about 1.0 V to the 20th cycle. As for other phenothiazine dyes, this peak can be attributed to the formation of di-cation initiating the polymerization of the monomeric dye [15]. Indeed, if the cyclic voltammograms were recorded in the range from −0.3 to 0.5 V, no changes in the position and height of reversible peaks were observed indicating no polymerization of the monomers. Moreover, transfer of the electrode in a fresh PB with no monomers resulted in sharp decrease of the above peaks down to the background currents typical for supporting electrolyte. Thus, the adsorption of monomeric dyes on bare GCE is rather weak and reversible and cannot result in reproducible peaks on appropriate voltammograms. After four cycles of the potential, two peaks remained on the voltammogram (*E_pc_* = −0.07 V and *E_pa_* = −0.27 V), in which the height slowly increased in the following cycling of the potential. 

It is interesting to compare the peaks with those of individual dyes in similar experimental conditions. Previously we reported about electropolymerization of Azure B and proflavine taken alone in the same range of potentials. Azure B was oxidized in 0.1 M HEPES, pH = 6.9, with formation of the broaden peak pair at −0.11 and −0.09 V and of one irreversible anodic peak at the potentials higher than 0.8 V [29]. However, the Azure B peak pair at low potentials remained on voltammograms during the whole period of potential cycling (20 cycles) whereas the anodic irreversible peak was decreased with the number of cycles similarly to that observed here for the mixture of dyes. Regarding proflavine [31], it formed quasi-reversible pair of the peaks with the peak potential difference (0.22 and −0.13 V) higher than that discussed above for the Azure B polymer and the Azure B–proflavine copolymer. In this pair, cathodic peak was slowly increased with the number of potential cycles and anodic one was quite stable. The behavior of irreversible anodic peak initiating polymerization was similar to that reported for individual dyes polymerized in similar conditions. 

The comparison of the voltammograms obtained earlier for individual dyes and that, in their mixture, made it possible to conclude that the polymerization starts with the Azure B, which anodic peaks dominated in first 2–3 cycles. The implementation of proflavine became significant in the next cycles and resulted in general simplification of the voltammograms and in the shift of the peaks in the low potential range to higher potentials. The coverage of the surface with the polymer was mainly finished after the fourth cycle. The following deposition of the polymer increased the thickness of the layer and suppressed the access of both monomers to the electrode surface. As a result, the electron exchange became less effective and the equilibrium potential was slightly shifted to its higher value. The involvement of both monomers is confirmed by appropriate peak potentials and existence of two peaks referred to individual products at first cycles of the potential.

The resulting film is rather stable and exerts redox activity being transferred in a fresh PB with no monomers (Figure 1b). On this voltammogram, the anodic peak (0.28 V) is closer to that of the proflavine and the cathodic peak (−0.08 V) to that of poly(Azure B). The voltammograms of all three reactants, i.e., Azure B, proflavine, and their mixture, are rather close to each other so that variation in the molar ratio of the dyes in the mixture did not result in quantitative changes of appropriate voltammograms. The ratio used in this work corresponded to the maximal difference in the peaks related to individual dyes and their mixture. The only conclusion was that lower quantities of Azure B decreased the relative height of the peak *a_1_* at first scan. This confirms the attribution of the peak to this monomer.

**Figure 2:**
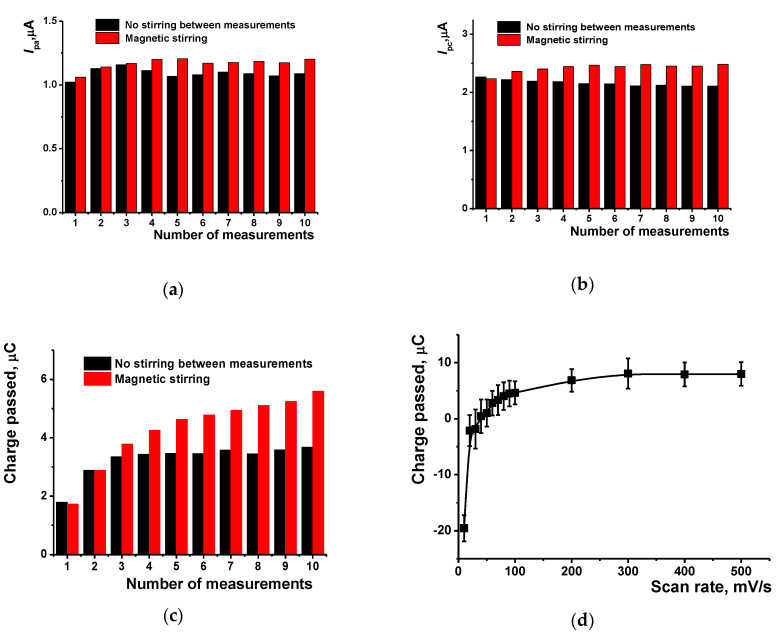
The dependency of cathodic (**a**), anodic (**b**) peak currents and charge passed (**c**) on the number of consecutive measurements separated by magnetic stirring of solution or by its equalization in open circuit mode. 0.025 M PB, pH = 7.0, scan rate 100 mV/s; (**d**) The dependence of the charge passed in the whole potential cycle between −0.4 and 0.8 V on the scan rate.

The stability of the coating was confirmed in the series of voltammetric measurements with individual sensors in the PB with no monomers. The range of the potential scan (from −0.4 to 0.8 V) excluded polymerization of the monomers that could be entrapped in the surface film. Contrary to electropolymerization, the runs of the potential were separated by a certain period of switched-off voltage (5 min.), in which the solution was magnetically stirred. The anodic peak current was stabilized after the 2–3 cycle, whereas the cathodic current with no stirring was constant starting from the first run (Figure 2b). 

In addition to the peak currents, the charge passed was determined by integration of the current–voltage curves. As could be seen from the Figure 2c, the charge passed was stabilized to the third measurement with no stirring. However, magnetic stirring resulted in a regular increase of the charge during the whole measurement series. This might be due to the elimination of small particles of the coating that are weakly attached to the surface and leave them between the measurements. This would increase the specific surface of the electrode interface. The charge passed also tends to increase with the scan rate to the limit similar to that reached in consecutive potential cycling (compare Figure 2c,d). At a low scan rate, oxidized forms of the dyes preferably determine the charge passed, which is negative and corresponds to a predominantly cathodic process of the oxidized dyes reduction. This coincides well with the fact that the dyes are commonly present in the oxidized (salt) form in aqueous solutions containing dissolved oxygen. However, at a scan rate higher than 40 mV/s, the charge passed becomes positive indicating the prevalence of the anodic reactions over the cathodic ones. This can result from the formation of rather dense film and low rate of electron exchange in the polymer layer. In such conditions, changes in the overall charge passed are less compensated for by the movements of the negatively-charged counter ions and, hence, less affect the charge passed in the cathodic branch of the voltammogram.

The electrode modified with the Azure B–proflavine copolymer was tested after stabilization at various scan rates to determine the nature of the rate-limiting stage. In the bilogarithmic plots, the slope of the linear graph was found in-between 0.5 (surface confined reactions) and 1.0 (diffusion limitation) [38] in the range from 10 to 500 mV/s. Appropriate regression equations are presented in Equations (1) and (2). Here, *I_pa_* and *I_pc_* are the anodic and cathodic peak currents, respectively, and ν is the potential scan rate.

Anodic peak current: log(*I_pa_*, μA) = (0.77 ± 0.01) + (0.67 ± 0.01) × log(*ν*, mV/s), *R*^2^ = 0.9961, *n* = 14 (1)

Cathodic peak current: log(*I_pc_*, μA) = (1.04 ± 0.01)+ (0.64 ± 0.01) × log(*ν*, mV/s), *R*^2^ = 0.9946, *n* = 14 (2)

The dependence of the peak currents on the scan rate was investigated in the absence of the monomers in solution so that diffusional transfer of redox species was impossible. In these conditions, the decrease of the slope against the value typical for the surface reactions (1.0) can be referred to the influence of the counter ions transfer mentioned above or to the slow electron exchange between the oxidized and reduced monomer items within the layer. Similar behavior was found earlier for the polymeric Neutral red [39]. The transfer coefficient α = 0.83 was determined using Laviron’s theory from the dependence of the peak potential on the scan rate (Equation (3)) [40,41]:

(3) $$E_{pc}=E^{0’}+\frac{RT}{\alpha nF}\ln\frac{RTk_{et}}{\alpha nF}-\frac{RT}{\alpha nF}\ln v$$

Here, *E*^0’^ is the formal redox potential, α is the transfer coefficient, *n* is the number of the electrons transferred, *F* is the Faraday constant, *R* is the universal gas constant, *T* is the temperature, K, and *k_el_* is the heterogeneous constant of the electron transfer.

### 3.2. The Comparison of the Redox Properties of Poly(Azure B), Poly(proflavine) and Their Copolymer

Although the behavior of the dyes used was very similar to each other, there is a difference, which can be related to the intrinsic processes of the electron exchange and counter ions transfer. To simplify the consideration, some of the changes observed are summarized in the Table 1 based on this work and our previous investigations performed in similar experimental conditions.

Among other properties, the pH dependence of the equilibrium potential is most sensitive to the monomer mixing. All three coatings mentioned in Table 1 exert a linear response toward pH according to the Nernst equation with the slopes of 29 mV/pH for poly(Azure B) and 59 mV/pH for the poly(proflavine) that correspond to the transfer of one H^+^ ion, two electrons, and two H^+^, two electrons, respectively. In case of the copolymer, the pH dependence is more complicated. Cathodic peak potential shows the pH dependence similar to that of Azure B (slope 53 mV/pH), whereas the anodic dependence formally corresponds to the transfer of 1.5 H^+^ ions per electron. Appropriate slope (43 mV/pH) is in between those obtained for the poly(Azure B) and poly(proflavine). In the same dependencies involving equilibrium potential corresponded to the half-sum of peak potentials, the copolymer showed maximal range of linearity (pH from 2.0 to 9.0), whereas the poly(proflavine) changed this potential only in basic media (pH from 6.0 to 9.0) and that of poly(Azure B) in weakly acidic conditions (pH from 3.0 to 6.0). It can be concluded that the copolymer combines the pH sensitivity areas of individual dyes and exerts better reversibility of electron/H^+^ exchange. The nominal transfer of 1.5 H^+^ per electron can be explained by simultaneous redox reactions of both monomers resulting in averaging of the stoichiometry of equilibrium.

### 3.3. DNA Deposition on the Copolymer of Azure B and Proflavine

Polyelectrolyte complexes of the redox active polymers and DNA have been successfully applied for the detection of specific biochemical interactions of the DNA influencing redox equilibria of the support [3]. For this purpose, polyaniline [42], poly(Neutral red) [27,43], poly(Methylene blue) [43], poly(Methylene Green) [43], poly(Azure B) [29], and poly(proflavine) [31] have been used. In this work, the DNA solution was applied on the working surface of the electrode covered with the copolymer and either dried or left capped with plastic tube preventing drying for a certain time. In voltammetric experiments, the contact with the DNA resulted in 10–15% decrease of the oxidation peak current irrespective of the DNA quantities and application protocol. However, incubation resulted in much higher deviation of the signal. For this reason, drying DNA solution was used in other experiments described. Taking into account low sensitivity of the voltammetry to such changes of the surface layer, the deposition of DNA was confirmed by SEM and EIS.

#### 3.3.1. SEM Monitoring of the Surface Layer Assembling

**Figure 3:**
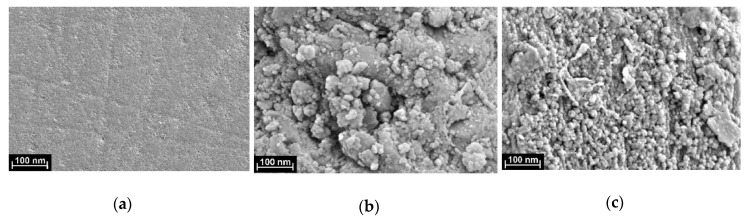
SEM microimages of bare glassy carbon electrode (**a**), of the copolymer of Azure B and proflavine prior to (**b**) and after DNA adsorption (**c**).

Figure 3 represents the morphology of the electrode surface on the stages of the electropolymerization and the DNA casting.

Polished glassy carbon showed smoothen surface with randomly positioned mechanical scratches. Deposition of the copolymer resulted in the formation of rough surface covered with the angular fragments. Their main fraction (60%) has the size of 40–55 nm. In comparison with individual dyes, which electropolymerization was studied earlier, the surface morphology is similar to that of proflavine. The latter one also forms aggregates covered with roundish particles [29], whereas poly(Azure B) had a uniform structure with elongated parallel inclusions [31]. 

Deposition of the DNA onto the copolymer surface changed its morphology. First, angular fragments distinguishable on the surface folds, disappear. Instead of them, well-defined roundish DNA aggregates with diameters of about 25–30 nm fill the hollows and the surface of the film. They are much better discernible than those described on the poly(proflavine) film and are about twice smaller.

Disappearance of the fragments visible on the copolymer film prior to the DNA application can be referred to their leaching from the surface to the solution. Negatively charged DNA molecules promote such a leaching for the weakly attached particles due to neutralization of their positive charge by the negatively-charged phosphate groups of the DNA skeleton. Different aggregation of the DNA molecules adsorbed on the surface of the poly(proflavine) and of the copolymer of Azure B and proflavine can be explained by the difference in the specific surface charge and in the hydrophobicity of both coatings.

#### 3.3.2. EIS Measurements 

**Figure 4:**
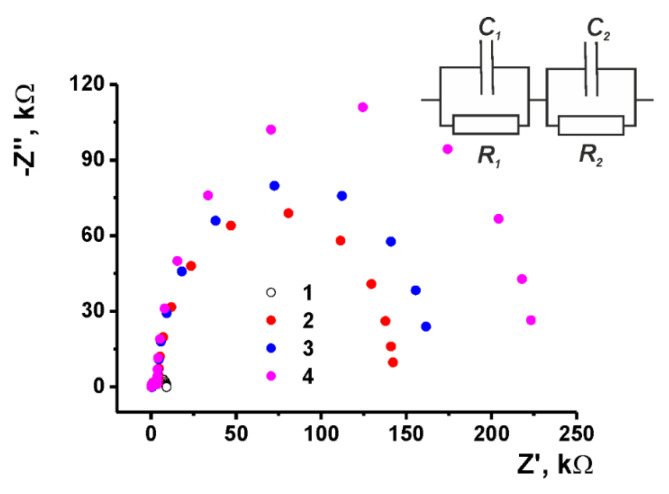
The Nyquist diagram obtained with the glassy carbon electrode (1) covered with poly(Azure B–proflavine) prior to (2) and after (3) application of DNA (2 μL of 1.0 mg/mL solution in 0.1 M HEPES + 0.03 M NaCl, pH = 7.0) or DNA-1.0 nM doxorubicin aliquot (4). Measurements in 0.025 M PB + 0.1 M KCl, pH = 7.0, in the presence of 10 mM [Fe(CN)_6_]^3−/4−^. Inset: equivalent circuit, *C*—constant phase element, *R*—charge transfer resistance.

EIS is a powerful tool of electrochemistry that is used for investigation of the charge transfer and for the monitoring of the interactions influencing the efficiency of electron exchange on the electrode interface. The Nyquist diagrams were obtained using 10 mM [Fe(CN)_6_]^3−/4−^ as redox probe at the mid-point potential calculated as the half-sum of the peak potentials (Figure 4). Here, *R* is the charge transfer resistance and *C* constant phase element, indices *1* and *2* correspond to the internal and external interface of the electrode-polymer layer. Roughness coefficient *n* specifying a non-ideal capacitance behavior of the constant phase element was found to be 0.78–0.90. Figure 3 represents the morphology of the electrode surface on the stages of the electropolymerization/DNA casting.

The Nyquist diagram contains two semi-circles corresponding to the electrode–film (smaller one) and film–solution (larger one) interfaces. Most changes observed during the surface layer assembling related to the charge transfer resistance of the outer interface. This coincides well with the accessibility of this interface to the reactant addition and hence higher sensitivity to various stages of the layer assembling in comparison with the inner interface electrode—polymer, which remains about constant within the whole series of measurements.

Electropolymerization of the Azure B and proflavine increased the *R_2_* value from 4 ± 1 to 130 ± 5 kΩ. Such changes can be attributed either to lesser electrostatic attraction of the oppositely-charged redox probe and cationic surface or to the denser coating (lower diffusion coefficient) of the ferricyanide ions penetrating the film. The increase in the number of the potential cycles and anodic polarization at the stage of the electrode modification did not significantly alter this value. This makes it possible to conclude that slower diffusion appeared to be more important than electrostatic interactions. The following application of the DNA molecules increased the *R_2_* value to 170 ± 3 kΩ. It is interesting to note that the treatment of the DNA with doxorubicin, an anthracycline dye intercalating the DNA helix, increased this value to 215 ± 8 kΩ. The capacitance remains about constant during the DNA application and doxorubicin introduction. Its small value (about 1 μF) confirms a low charge separation on the electrode interface and the suggestion about the predominant influence of the diffusion factors on the EIS parameters. Doxorubicin molecules partially compensate for the negative charge of the phosphate residues in the DNA backbone and make weaker electrostatic interactions between them and the [Fe(CN)_6_]^3−/4−^ ions of the redox probe.

Thus, the modification protocol used provides the deposition of the polymeric film and adsorption of the DNA molecules, which affect the behavior of the sensor due to the variation of the permeability of the surface layer for small ions of redox probe.

### 3.4. Determination of Doxorubicin

#### 3.4.1. Doxorubicin Oxidation on Glassy Carbon Prior to and After the Electropolymerization Stage

**Figure 5:**
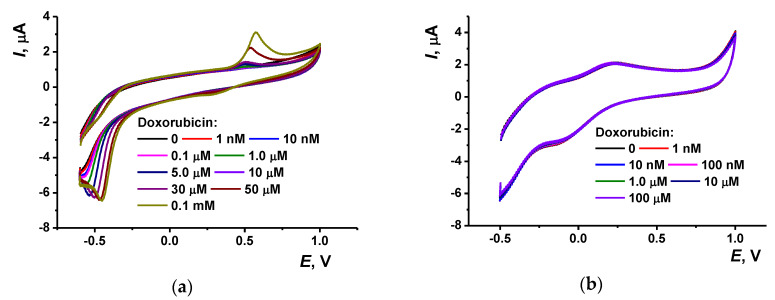
Cyclic voltammograms of doxorubicin recorded on glassy carbon, bare (**a**) and modified with copolymer of Azure B and proflavine (**b**). 0.025 M PB + 0.1 M KCl, pH = 7.0, scan rate 100 mV/s.

In accordance with the literature data [44,45], hydroquinone and benzoquinone units of doxorubicin are involved in electrode reaction with formation of separated signals. The largest one is commonly observed at negative potentials (from −0.5 to 0.65 V depending on the electrode material) and another one, much lower, at positive potential (0.4–0.6 V). This information coincides well with the results obtained on bare GCE (Figure 5). 

With no modifier, an irreversible cathodic peak at −0.45 V and one irreversible anodic peak at 0.55 V appear on the voltammogram. They are regularly increasing with the doxorubicin concentration. However, after the electrode modification, doxorubicin does not affect the signals of the underlying copolymer within the concentration range of five orders of magnitude (Figure 5b). Probably, the molecules of doxorubicin cannot reach the electrode or compete with the electron exchange chain within the surface layer. This might result from hydrophobicity of the analyte or steric limitation of its adsorption on the polymer layer.

#### 3.4.2. Determination of Doxorubicin with DNA Sensor Based on Copolymer of Azure B and Proflavine

**Figure 6:**
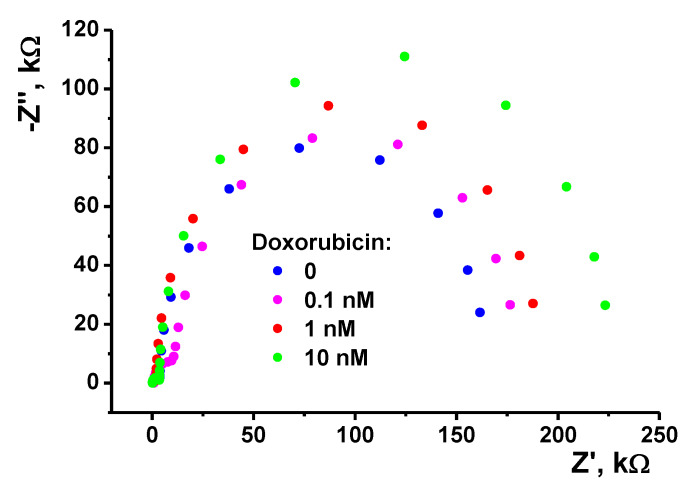
The Nyquist diagram obtained on the glassy carbon electrode covered with poly(Azure B–proflavine) and DNA previously mixed with doxorubicin solution (2 μL of the mixture containing 1.0 mg/mL DNA in 0.1 M HEPES + 0.03 M NaCl, pH = 7.0). Measurements in 0.025 M PB + 0.1 M KCl, pH = 7.0, in the presence of 10 mM [Fe(CN)_6_]^3−/4−^.

The investigations were continued in the EIS mode, which is more sensitive than cyclic voltammetry to the surface-confined reactions. Doxorubicin intercalates the DNA helix with inclusion between the pairs of nucleobases. This reaction changes the volume and conformation of the DNA molecules and partially compensates for the negative charge of the DNA and its ability for electrostatic attractions with the positively-charged moieties. However, incubation of the electrode covered with the copolymer Azure B–proflavine and adsorbed DNA did not result in an increase of the charge transfer resistance. Contrary to that, the *R_2_* value decreased to the level typical for the copolymer prior to the DNA application. This might be due to the lesser repulsion of the [Fe(CN)_6_]^3−/4−^. For this reason, the following experiments were performed with preliminary treatment of the DNA with the doxorubicin solution performed prior to the DNA application on the electrode surface. After 20 min. of incubation, the mixture was applied on the electrode covered with the copolymer. After drying, the electrode was carefully washed with deionized water and then immersed in the working PB for EIS measurements. As was mentioned previously, all the changes of the surface layer affected mostly the impedimetric parameters on outer interface. The charge transfer resistance linearly increased with the doxorubicin concentration in the range from 10 nM to 0.03 nM. Figure 6 shows appropriate changes of the Nyquist diagram.

Thus, the sensitivity of the EIS parameters toward doxorubicin was much higher than that of voltammetry. The linear range of the concentrations determined was 3 × 10^−11^–1 × 10^−8^ M (calibration Equation (4)):

*R_ct_*, kΩ = (380 ± 12) + (21 ± 1) log(*c*, M), *R*^2^ = 0.9673, *n* = 5 (4)

In the same concentration range, the capacitance of the outer interface increases with the doxorubicin concentration from 1.3 to 1.9 μF and remains constant after reaching 1 nM concentration. The limit of detection (LOD) corresponded to the S/N = 3 criterion was found to be 1 × 10^−11^ M. These characteristics are similar or better than those of other electrochemical sensors and DNA sensors described for doxorubicin determination. The comparison of the characteristics of such sensors is summarized in Table 2.

The only DNA sensor that showed higher sensitivity was comprised of the polyaniline with the physically adsorbed DNA. The advantage in its characteristics can be referred to a higher level of the redox activity of the polymer and higher sensitivity of its redox properties toward microenvironment than those of polyphenothiazines. Meanwhile, the electropolymerization of aniline requires strongly acidic media and is sensitive to the presence of the oxygen that can partially suppress the electroconductivity of the polymer formed. For this reason, only freshly distilled aniline is used in the electropolymerization. The incubation performed at pH 3.0 can also negatively affect the results if complex media is analyzed. 

For the selectivity assessment, the impedimetric signal of 1.0 nM doxorubicin was measured in the presence of 10 μM bovine serum albumin and 1.0 μM sulfamethoxazole. The variation of the charge transfer resistance averaged for six individual DNA sensors did not exceed 6%. This coincides well with the results obtained earlier for the polyaniline based DNA sensors with impedimetric and voltammetric signals [3,49].

The results obtained make it possible to conclude that impedimetric measurements allow for the sensitive determination of doxorubicin in the sub-nanomolar range of its concentrations. 

#### 3.4.3. Measurement Precision and DNA Sensor Lifetime

Sensor-to-sensor repeatability calculated from the EIS data was equal to 5.5% (six individual sensors, 1.0 nM doxorubicin). Each sensor was used only once because of irregular changes in the sensitivity observed in the next attempts of the signal determination. The GCE covered with the copolymer of the Azure B and proflavine can be stored in dry conditions at 4 °C for at least six months. In the following DNA application and doxorubicin signal measurement, the deviation tends to increase to 10% toward the end of the storage period. 

#### 3.4.4. Real Sample Analysis

The DNA sensor developed was tested in the determination of the doxorubicin content in two medications, i.e., Doxorubicin-TEVA^®^ and Doxorubicin-LANS^®^ ((lyophilizates for intravascular injection solutions). In both cases, the medications were dissolved first in deionized water and then in working buffer solution and mixed in 9:1 ratio with 10.0 mg/mL DNA to final nominal concentration of 1.0 mg/mL (see Section 2.4 for details). Then the mixture was applied on the electrode surface and dried. Unbounded components were carefully removed with deionized water and PB and the EIS measurements were performed as described above. The recovery was assessed using the calibration TEVA, plot of the drug obtained in buffered media with standard drug solutions. For the doxorubicin- the recovery was equal to 99 ± 7% (six measurements) and that for the doxorubicin-LANS 102 ± 10%. It should be mentioned that both preparations contained stabilizers (lactose and mannite, respectively). It is probably that the low activity of the polymer coating to oxidizable species showed, for doxorubicin itself, suppressed its influence on the biosensor signal.

In a similar manner, the influence of serum proteins (for the example of bovine serum protein, see Section 3.4.3) and plasma electrolytes (Ringer–Locke’s solution) was estimated with 1 nM doxorubicin solution. The recoveries of 106 ± 8% and 102 ± 12% were found. Certainly, more attention should be paid to the assessment of the factors that affect the biosensor response with real blood samples. However, on this stage of “proof-of-concept” it can be concluded that the DNA sensor developed can find application for preliminary assessment of the concentration of doxorubicin in patients’ serum after further validation.

## 4. Discussion

The electropolymerization of an equimolar mixture of the Azure B and proflavine made it possible to modify the electrode with a layer that provides both reliable adsorption of the native DNA and determination of its interaction with anthracycline dye. Contrary to similar protocols previously elaborated for individual dyes, the mixed composition offered some advantages, i.e., a denser and durable film with a rough surface capable of electron exchange and DNA implementation. The size distribution of the polymer and DNA adsorbed was different from the polymeric dyes described elsewhere by lower size of the DNA aggregates and their narrow distribution. The comparison of the cyclic voltammograms and the SEM images with those of poly(Azure B) and poly(proflavine) showed the participation of both components in the electron exchange and related H^+^ transfer. 

Doxorubicin is one of the anthracycline antibiotics active against solid tumors and hematological malignancies [52]. Its application is limited by rather high cardiotoxicity [53]. For this reason, its monitoring in the biological fluids and preparations is extremely important in chemotherapy for individual dose establishment. Additionally, doxorubicin has found a broad application as a standard intercalator in investigations of electrochemical DNA sensors. In addition to intercalation it is involved in the generation of reactive oxygen species that damage DNA and produce 8-oxoguanine as an indicator of oxidative damage [54]. For this reason, new systems of doxorubicin determination based on polymer-DNA films exerting own redox activity are important for both pharmaceutical applications and DNA biosensors progress.

For EIS measurements of DNA sensors, an [Fe(CN)_6_]^3−/4−^ redox probe is mostly used. Due to the negative charge and repulsion from phosphate residues of the DNA backbone, the impedimetric signal becomes sensitive to any biochemical interactions that take place on the electrode interface with DNA molecules. Two main mechanisms are mostly considered to explain changes in the charge transfer resistance of these biosensors contacted with target analytes, i.e., (i) changes in electrostatic interactions caused by shielding phosphate groups of the DNA, and (ii) changes in the permeability of the surface coating for small ions due to denser packing and deposition of non-conductive molecules interacting with DNA [54]. 

Considering the influence of doxorubicin on EIS parameters, one of the unexpected results was that electrostatic interactions mentioned played less significant role in the signal generation than usually for other DNA sensors based on the redox active polymers. Variation of the permeability of the surface layer for small ions (ferricyanide as redox probe) is most important factor explaining the performance of the biosensor. Changes in the DNA aggregation on the polymer film initiated by an intercalator could also suppress the transfer of the redox probe to the electrode but SEM data did not allow quantifying changes in the aggregation on the images.

Although the copolymer synthesized showed reversible redox behavior, its activity was found to be too small for effective participation in the electron transfer to the diffusionally-free reactants. The attempts to determine doxorubicin by its mediated oxidation on the modified electrode showed only minor variation of the current. However, this low activity can be considered as an advantage if the biosensor is applied for drug testing. Indeed, medications contain antioxidants to stabilize drugs and increase the drug storage period. In this work, no influence of such stabilizers was found for two different species (mannite and lactose). This makes biosensor also attractive for the drug residues detection in biological liquids with minimal sample treatment. Summarizing the specific properties of the DNA sensor, one could suppose, hydrophobicity of the electrode interface can be critical factor affecting the biosensor behavior and its high sensitivity toward doxorubicin.

## Figures and Tables

**Table 1:** The comparison of the properties of electropolymerized coatings of the phenazine dyes obtained by multiple cycling of the potential.

Property	Poly(Azure B) [29]	Poly(proflavine)	Copolymer [31]
Stability of redox parameters	The currents regularly decrease in consecutive measurements	Stable	Stable to 2–3 cycle
log*I_p_*-logν slope	Oxidation: 1.1 (polymer), 0.83 (monomer)	Oxidation: 0.81	Oxidation: 0.67
	Reduction: 0.96 (monomer)	Reduction: 0.64	Reduction: 0.64
Transmission coefficient	0.56 (polymer)	0.57	0.83

**Table 2:** Analytical characteristics of the determination of doxorubicin with electrochemical sensors and DNA sensors.

Surface Layer/Electrode	Concentration Range	LOD, nM	Ref
Electrochemical sensors			
Carbon nanotubes	20–500 nM	-	[46]
Ionic liquid/ZnO in carbon paste	0.07–5000 μM	9.0	[47]
Mesoporous carbon nanospheres/reduced graphene oxide	10 nM–10 μM	1.5	[44]
Basal plane pyrographite	0.01–1 μM	10	[45]
Fe_2_Ni@Au/reduced graphene oxide	5.5–9.2 μM	1460	[48]
Electrochemical DNA sensors			
Poly(Azure B)/DNA	0.1 μM–0.1 nM	0.07	[29]
Poly(proflavine)/DNA	1 nM–0.1 μM	0.3	[31]
Polyaniline/DNA	1.0 pM–1 mM	0.0006	[49]
Carbon nanotubes/polylysine/DNA	2.5 nM–0.25 μM	1.0	[50]
Poly(Neutral red)/DNA	0.01–100 μM	0.1	[51]
Poly(Azure B–proflavine)	0.03–10 nM	0.01	This work
